# Parental attitudes and perceptions associated with childhood vaccine exemptions in high-exemption schools

**DOI:** 10.1371/journal.pone.0198655

**Published:** 2018-06-14

**Authors:** Heidi L. Pottinger, Elizabeth T. Jacobs, Steven D. Haenchen, Kacey C. Ernst

**Affiliations:** 1 Mel and Enid Zuckerman College of Public Health, University of Arizona, Tucson, Arizona, United States of America; 2 University of Arizona Cancer Center, University of Arizona, Tucson, Arizona, United States of America; ESIC Medical College & PGIMSR, INDIA

## Abstract

Previous work demonstrates that individuals who obtain exemptions from school immunization requirements are geographically clustered, making regional differences in vaccination coverage a significant concern. Even where exemption levels are high, there are still parents that vaccinate. School-level assessments have determined that exemptors are more likely to attend wealthier schools with fewer minorities. Few studies have assessed divergent opinions within the context of a higher-exemption community to examine subtle differences in opinion surrounding vaccinations. Therefore, the objective of this work was to assess attitudes and perceptions towards vaccinations and compare them for exemptors and non-exemptors. We administered surveys to parents in high-exemption (>10%) elementary schools in Arizona during the 2012–13 school year. A total of 404 surveys were completed by parents among schools in Maricopa (n = 7) and Yavapai (n = 2) counties. Of these, 35% (n = 141) were exemptors and 65% (n = 261) were non-exemptors. Exemptors were more likely than non-exemptors to be concerned about serious side-effects (p<0.001). They were more likely to report knowing someone who had been diagnosed with a vaccine-preventable disease (p<0.001) but less likely to report that this had been a serious illness in that person (p<0.001) and they believed it is better for a child to develop immunity through illness than vaccination (p<0.001). They were less likely to trust physicians (p<0.001) and information about vaccines (p<0.001) and were more likely to obtain their health care from a naturopath (p<0.001). In summary, exemptors in these Arizona schools do not appear to be exempting their children from vaccinations due to convenience, as has been hypothesized in other settings. Based on the divergent views within high-exemption schools and reported distrust of the medical establishment, target interventions for high-exemption schools are discussed. Additionally, given the lack of effective non-policy based interventions to-date, the negligible declines in personal belief exemption rates, and vaccine preventable disease rate increases in Arizona, especially in high-exemption areas, legislative action in Arizona may also warrant further investigation.

## Introduction

Vaccine exemptions are currently an area of intense interest in public health, particularly in light of the measles outbreaks in 2015 and 2016 [1]. Over the last century, universal recommendations for vaccination in the United States (US) have substantially reduced the morbidity and mortality from vaccine-preventable diseases (VPDs) [2]. While there can be rare adverse events after vaccination, the Institute of Medicine (IOM) unequivocally reported in 2011 that the benefits far outweigh any risk [3]. In 2013, the Institute of Medicine (IOM) reported that a growing trend of delaying vaccinations or exempting from them altogether has contributed to increases in vaccine-preventable outbreaks and mortality in the US [4].

In the US, all states and the District of Columbia have laws requiring vaccinations for entry into public schools; however, approximately 20 states allow parents to waive vaccines based on personal beliefs. The protocol for obtaining these personal belief exemptions (PBEs) varies widely by state [5,6]. More comprehensive exemption procedures are associated with fewer exemptions [5] and ease of PBE has been associated with decreased coverage in immunization [6], as well as increased incidence of pertussis [6,7] and measles [8,9]. Most recently, clusters of pertussis cases have been shown to be associated with clusters of vaccine refusal, even after adjusting for demographic factors [10]. Arizona is one of approximately 15 states in which an individual can obtain an exemption with a simple form and parent signature [6,11].

Among states reporting the highest exemption rates in the US, Arizona ranked eighth with 4.9% (n = 4,195) of Arizona kindergarteners having reported PBEs during the 2013–14 school year, an increase of 0.7% from 2012–2013 [12]. While the most recent state-wide PBE rate reports a decline of 0.1% from the previous year, overall the rate of vaccine exemptions has tripled in Arizona in the past decade [12,13]. In the most populous county, Maricopa County, 2014–15 exemption rates for kindergartners were reported at 5.1% with Yavapai County reporting the highest PBE rate in the state (10.0%) [13]. Reported cases of vaccine-preventable diseases (VPD) in Arizona have also increased. VPD have more than quadrupled in Arizona from 2008 (n = 358) through 2013 (n = 1,568), representing an average increase reported of 242 cases/year. Specifically, cases of pertussis have exhibited a six-fold increase over this time period [14]. In 2013, low vaccination rates in a small community in northwest Arizona were cited as a likely explanation for a large outbreak impacting this isolated community, accounting for nearly half the state’s cases that year [14]. Even after excluding outbreak-related cases from 2013, there was still a four-fold increase in VPD from 2008 to 2013.

Regional clusters of high levels of permanent personal belief vaccine exemptions (>10%) have been identified in Arizona and are associated with attendance at schools that have higher wealth indicators and a lower proportion of minorities [15,16]. Reasons for vaccination hesitation vary, as do immunization patterns, and are influenced by factors including confidence, convenience, and complacency [17,18]. Vaccine hesitancy varies not only across vaccines but also across time and place [18]. As infectious disease transmission occurs in schools and vaccine hesitancy is context and place-specific, it is critical to determine the attitudes and perceptions of parents who opt to exempt or delay their children from vaccination in order to better understand how to enact effective interventions. A cross-sectional survey was conducted among parents whose children attended a school located within high-PBE geographic regions, with the goal of ascertaining differences in attitudes and perceptions about vaccines between responding non-exemptors and exemptors.

## Methods

This study was reviewed by the University of Arizona and Arizona Department of Public Health Institutional Review Boards, as well as by applicable Maricopa and Yavapai Counties and public school systems. The study was determined to be ethical and of minimal risk.

### Identification of study sites

Previous work identified high-exemption regions in Maricopa and Yavapai counties [15]. Data for PBE rates was obtained from the 2010–11 Immunization Data Reports from Arizona schools based on availability. The permanent PBE rate for kindergarteners in reporting Arizona schools was derived by dividing the number of children with permanent exemptions by the total number of children enrolled in kindergarten. Schools in Maricopa and Yavapai Counties with PBE rates greater than 10 percent were noted.

### Recruitment

District nurses and/or administrators presiding over elementary schools identified to have higher exemption rates (>10%) were sent a recruitment e-mail to request their participation in this study. If they agreed, the required research request documentation for each respective district was completed. Each participating school received $200 to be utilized for their nursing and healthcare needs and to offset the costs of study-related tasks. Nine schools agreed to participate in the study—seven were from Maricopa County, which includes the Phoenix metropolitan area; and two were from Yavapai County, which includes Prescott, AZ and parts of Sedona, AZ. Parents were recruited by their respective school’s central office to complete surveys during August 2013 and were compensated with a five-dollar gift card for their time.

### Survey development, distribution & data analysis

The survey was adapted from the Parent Attitudes about Childhood Vaccines (PACV) survey, a valid and reliable instrument to identify vaccine-hesitant parents. The PACV survey utilizes three different response formats: dichotomous, 5-point Likert scale, and an 11-point scale [19,20]. For this study, we also included parent socio-demographic items, questions concerning parental motivation and method for obtaining exemptions, as well as questions regarding VPD history. Survey drafts were administered to a group of approximately ten volunteers to identify lack of clarity in questions, time to take the survey, and survey flow. Modifications were made on the feedback from these individuals. School administrators were sent either paper or electronic surveys (published using SurveyMonkey software) to distribute to parents school-wide depending on the preference of school administrators. Surveys were available in both English and Spanish (S1 File). Seven schools (Maricopa County) preferred to distribute the surveys electronically while two schools (Yavapai County) preferred paper-based surveys.

An IRB-approved letter to parents describing the study purpose, minimal risk, indirect benefits of participation, and contact information for study investigators and human subject’s protection in both English and Spanish preceded the survey link or paper-based survey. Upon completion of the electronic survey, parents were directed to a separate link to enter contact information to receive a gift card. Administrators distributing paper-based surveys managed a list of participants which was submitted after data-entry. Parents were informed that this information would not be linked to survey responses. Survey response rate was calculated using total enrollment provided by school administrators at the time of survey distribution (where available) as well as publicly-available data from the National Center of Education Statistics [21].

Respondents were categorized as either “exemptors” or “non-exemptors” based on parental self-report of exemption status in the survey. Differences in proportions were calculated for dichotomous categorical variables between the two groups using a Wilcoxon rank sum test. For continuous variables, a two-tailed Student’s t-test was employed to ascertain differences in means. For categorically ranked variables with more than one category, chi-square analyses were conducted. All analyses were conducted using SAS v. 9.3 [Cary, NC], and Stata v. 13 [College Station, TX].

## Results

Of the 27 schools meeting inclusion criteria, 15 responded and nine agreed to participate in the study, for a response rate of 33.3%. The mean kindergarten permanent PBE rate, based on 2010–11 data for these nine schools, was 18.5%. Among the participating schools, surveys reached approximately 2800 households via email or paper copy, with responses received from 404 parents (S1 Data), for a response rate of 14.4%. The mean age of respondents was 37.2 ±6.3 years and the average number of children the parents reported having was 2.7±1.3 children (Table 1). The majority of respondents were female (85.0%); white (87.6%); married (82.9%); held a Bachelor’s degree or higher (55%); had private insurance (74.0%); visited an M.D. (83.1%); were employed (63.0%) and/or had a partner who was employed (84.0%); and earned a household income greater than or equal to $50,000 annually (75.5%; Table 1).

**Table 1:** Characteristics of survey respondents^1^.

Characteristics	n (%)^1^
	N = 404
Female	334 (85.0)
Mean age (SD)	37.2 (6.3)
**Race**	
White	339 (87.6)
Black/African American	7 (1.8)
Asian/Pacific-islander	6 (1.6)
Hispanic	19 (4.9)
Other	16 (4.1)
**Marital Status**	
Married	324 (82.9)
Never Married	18 (4.6)
Not Married, Living with Partner	13 (3.3)
Divorced or Separated	36 (9.2)
**Highest Level of Education Completed**	
High school or less	21 (5.3)
Some college but no degree	93 (23.6)
Associate’s Degree	65 (16.5)
Bachelor’s Degree	127 (32.2)
Graduate or Professional Degree	88 (22.4)
**Insurance Status**	
Private Insurance	292 (74.0)
Public Insurance	78 (19.7)
Uninsured	25 (6.3)
**Type of Doctor Visited**	
M.D.	251 (83.1)
D.O.	30 (9.9)
Naturopath	11 (3.6)
No Doctor	10 (3.3)
**Employment Status**	
Self is employed	188 (63.0)
Partner is employed	253 (84.0)
**Total Household Income Range**	
$100,000 or greater	95 (25.3)
$75,000–99,999 |	85 (22.6)
$50,000–74,999	104 (27.6)
$35,000–49,999	50 (13.3)
Less than $35,000	42 (11.2)

^1^Totals for each category may not sum to total sample size due to non-response. Percentages represent percentages among those responding.

Note: The following categorical characteristics were not included due to lack of respondents: Race: American Indian/Alaskan Native; Marital Status: Widowed.

Table 2 presents respondent characteristics stratified by exemptors (n = 142; 35.0%) and non-exemptors (n = 262; 65.0%). There were no statistically significant differences between exemptors and non-exemptors for sex, age, race, marital status, number of children, education level, insurance or employment status, or income. However, respondents who indicated that they sought care from a Naturopath or a Doctor of Osteopathy were statistically significantly more likely to be exemptors (23.6%) than non-exemptors (7.8%; p<0.001).

**Table 2:** Differences in characteristics of exemptors vs. non-exemptors.

Characteristics	Exemptor n (%)^1^	Non-Exemptor n (%)^1^	P-value
	N = 142 (35%)	N = 262 (65%)	
Gender—Female	118 (86.0)	216 (85.0)	0.77^2^
Mean age (SD)	36.8 (6.1)	37.5 (6.4)	0.30^3^
**Race**			0.49^2^
White	122 (90.4)	216 (86.0)	
Black/African American	2 (1.5)	5 (2.0)	
Asian/Pacific-islander	2 (1.5)	4 (1.6)	
Hispanic	3 (2.2)	16 (6.4)	
Other	6 (4.4)	10 (4.0)	
**Marital Status**			0.5^2^
Married	111 (80.43)	212	
Never Married	5 (3.62)	13	
Not Married, Living with Partner	6 (4.35%)	7	
Divorced or Separated	16 (11.6%)	20	
**Highest Level of Education Completed**			0.14^2^
High school or less	5 (3.6)	12 (7.7)	
Some college but no degree	36 (26.1)	57 (22.4)	
Associate’s Degree	30 (21.7)	34 (1.3)	
Bachelor’s Degree	39 (28.3)	88 (34.5)	
Graduate or Professional Degree	28 (20.3)	60 (23.5)	
**Insurance Status**			0.18^2^
Private Insurance	101 (72.7)	190 (74.5)	
Public Insurance	25 (18.0)	53 (20.8)	
Uninsured	13 (9.3)	12 (4.7)	
**Type of Doctor Visited**			0.00^2^
M.D.	76 (69.1)	175 (91.1)	
D.O.	15 (13.6)	15 (7.8)	
Naturopath	11 (10)	0 (0)	
No Doctor	8 (7.3)	2 (1.04)	
**Employment Status**			
Self is employed	65 (59.1)	123 (65.1)	0.30^2^
Partner is employed	93 (85.3)	159 (83.7)	0.12^2^
**Total Household Income Range**			0.07^2^
$100,000 or greater	27 (20.5)	68 (27.9)	
$75,000–99,999 |	27 (20.5)	58 (23.8)	
$50,000–74,999	46 (34.8)	58 (23.8)	
$35,000–49,999	21 (15.9)	29 (11.9)	
Less than $35,000	11 (8.3)	31 (12.7)	

^1^Totals for each category may not sum to total sample size due to non-response. Percentages represent percentages among those responding.

^2^P-value calculated using Pearson’s chi-square test.

^3^P-value calculated using Two-tailed t-test.

Note: The following categorical characteristics were not included due to lack of respondents: Race: American Indian/Alaskan Native; Marital Status: Widowed.

As shown in Table 3, a total of 91 of 142 exemptors provided data for their motivation and method for obtaining an exemption. The majority (90.1%) indicated that their primary motivation for doing so was due to a personal belief and nearly 10% cited a reason of convenience. Furthermore, of exemptors who responded indicating the method by which they had obtained the non-medical exemption, the majority (85.0%) had procured the exemption form themselves; while 15.0% indicated that they were offered the form by their school’s office without having asked for it (Table 3).

**Table 3:** Primary reason for requesting a nonmedical exemption, and method (n = 91).

Survey Question and Answer Options	Exemptor n (%)
**If you have taken a non-medical exemption for your children’s shots, what was your primary reason?**	
I did not have time to go to the doctor to update shots	6 (6.6)
I do not believe my child should get shots	82 (90.1)
I lost my child’s shot records	3 (3.3)
**If you have taken a non-medical exemption for your children’s shots, how did you obtain it?**	
I researched and downloaded a form from the Internet	22 (23.7)
I requested the paperwork from the school office	57 (61.3)
The school office offered the form without me asking	14 (15.0)
I saw the forms sitting in the office	0 (0.0)

Table 4 presents a comparison of attitudes toward vaccines between exemptors (n = 141) and non-exemptors (n = 261). On a Likert-type scale from 0 (not at all sure) to 10 (completely sure), exemptors were significantly less likely to agree that the recommended shot schedule is a good idea for their child than non-exemptors, with scores of 2.3 (SD = 2.9) and 8.1 (SD = 2.3), respectively; p<0.001). On a scale from 0 (do not trust at all) to 10 (completely trust), exemptors were also significantly less likely to trust their child’s doctor than non-exemptors, with scores of 6.9 (SD = 2.5) and 8.6 (SD = 1.7), respectively (p<0.001). On a scale ranging from 1 (strongly disagree) to 5 (strongly agree), exemptors were more likely than non-exemptors to agree that children get more shots than are good for them with scores of 4.2 (SD = 1.1) and 2.4 (SD = 1.1), respectively (p<0.001), that it is better for children to get fewer shots at the same time [4.3 (SD = 0.9) and 3.2 (SD = 1.1), respectively; p<0.001), and that it is better for their child to develop immunity by getting sick than through immunization [3.4 (SD = 1.1) and 2.1 (SD = 1.0), respectively; p<0.001].

**Table 4:** Differences of perceptions and attitudes regarding vaccinations between exemptors and non-exemptors.

Survey Question/Statement and Answer Scale	Exemptor n = 141 (35.0%)	Non-Exemptor n = 261 (65.0%)	P-value
	Mean (SD)	Mean (SD)	
**How sure are you that following the recommended shot schedule is a good idea for your child?**			
Scale from 0 (Not at all sure) to 10 (Completely sure)	2.3 (2.9)	8.1 (2.3)	<0.001^2^
**All things considered, how much do you trust your child’s doctor?**			
Scale from 0 (Do not trust at all) to 10 (Completely trust)	6.9 (2.5)	8.6 (1.7)	<0.001^2^
**For all of the following**: Scale from 1 (Strongly disagree) to 5 (Strongly agree)			
**Children get more shots than are good for them**.	4.2 (1.1)	2.4 (1.1)	<0.001^2^
**I believe that many of the illnesses shots prevent are severe**.	3.4 (1.2)	4.3 (0.90)	<0.001^2^
**It is better for my child to develop immunity by getting sick than to get a shot**.	3.4 (1.1)	2.1(1.0)	<0.001^2^
**It is better for children to get fewer shots at the same time**.	4.3 (0.9)	3.2 (1.1)	<0.001^2^
**I trust the information I receive about shots**.	2.0 (1.1)	3.8 (0.9)	<0.001^2^
**I am able to openly discuss my concerns about shots with my child’s doctor**.	3.6 (1.4)	4.4 (0.8)	
**For all of the following**: Scale from 1 (Not at all concerned) to 5 (Extremely concerned)			
**How concerned are you that your child might have a serious side effect from a shot?**	4.3 (1.0)	3.0 (1.2)	<0.001^2^
**How concerned are you that any one of the childhood shots might not be safe?**	4.4 (0.9)	2.9 (1.2)	<0.001^2^
**How concerned are you that a shot might not prevent the disease?**	3.6 (1.3)	2.6 (1.2)	<0.001^2^
**Overall how hesitant about childhood shots would you consider yourself to be?**			
Not at all hesitant	3 (2.1)	104 (39.8)	
Not too hesitant	16 (11.3)	103 (39.5)	
Not sure	0 (0)	10 (3.8)	
Somewhat hesitant	50 (35.5)	38 (14.6)	
Very hesitant	72 (51.1)	6 (2.3)	<0.001^3^
**Do you know someone who has had a severe reaction to a shot?** (Yes answers presented)	84 (59.6)	55 (21.1)	
**If you had another infant would you want them to have all of their vaccinations?** (Yes answers presented)	26 (18.4)	229 (87.7)	<0.001^3^
**Do you know someone who had a disease they could have gotten a shot for?**	64 (45.4)	91 (34.9)	
Yes, they didn’t need a doctor	38 (27.0)	27 (10.3)	
Yes, they needed a doctor	11 (7.8)	37 (14.2)	
Yes, they were hospitalized	15 (10.6)	27 (10.3)	<0.001^3^

^1^Totals for each category may not sum to total sample size due to non-response. Percentages represent percentages among those responding.

^2^P<0.001, calculated using Wilcoxon rank-sum test (unless otherwise noted).

^3^P<0.001, calculated using Pearson’s chi-square test.

Non-exemptors were significantly more likely than exemptors to agree that many of the illnesses vaccines prevent are severe, with scores of 4.3 (SD = 0.90) and 3.4 (SD = 1.2), respectively (p<0.001), and that they are able to openly discuss their concerns about vaccines with their child’s doctor [4.4 (SD = 0.8) and 3.6 (SD = 1.4), respectively; p<0.001]. On a scale from 1 (not at all concerned) to 5 (extremely concerned), exemptors were also significantly more likely than non-exemptors to be concerned that their child might have a serious reaction from a vaccine [4.3 (SD = 1.0) and 3.0 (SD = 1.2), respectively; p<0.001), that childhood vaccines might not be safe [4.4 (SD = 0.9) and 2.9 (SD = 1.2), respectively; p<0.001), and that vaccines might not confer protection against the disease they are intended to prevent [3.6 (SD = 1.3) and 2.6 (SD = 1.2), respectively; p<0.001)]. The results in Table 4 also show that exemptors were statistically significantly more hesitant about childhood vaccinations compared to non-exemptors (p<0.001). Overall, 86.6% of responding exemptors were at least somewhat hesitant regarding childhood vaccinations, compared to 16.9% of non-exemptors (p<0.001). Finally, while a significantly greater proportion of exemptors (45.4%) compared to non-exemptors (34.9%) indicated that they knew someone who had a VPD, 27.0% of exemptors reported that the individual did not need a doctor compared to approximately 10.3% of non-exemptors (p<0.001).

## Discussion

The overall findings of the present study indicate that there are major differences between vaccine-exempting and non-exempting parents from Arizona schools located in regional clusters with high PBE rates. Compared to non-exemptors, exemptors were significantly more likely to visit a Naturopath or a D.O. rather than a M.D. They were also less likely trust medical professionals and information that they receive about vaccinations. In addition, exemptors were significantly more likely to be concerned about safety of vaccinations and to believe that children receive too many shots.

Previous studies have reported higher exemption rates in private schools compared to public schools, and in communities where higher proportions of the population are white, college-educated, and earn relatively higher incomes [16,22–24]. In Arizona, charter schools and those with low prevalence of free and reduced lunches have significantly higher rates of PBEs [15]. As anticipated, based on our restriction to high-exemption schools, there were no significant differences between the reported socio-demographic characteristics of responding exemptors and non-exemptors.

As summarized above, our findings indicated that parents who refused vaccinations were more likely to visit a Naturopath or a D.O. for medical care, confirming prior work among schools in Colorado, Massachusetts, Missouri, and Washington [25]. External factors, such as healthcare providers, can influence parental perceptions about disease risk and severity as well as their confidence in vaccines [17,26,27]. Therefore, it is critical to engage all healthcare providers. Further research is needed to identify, tailor, and evaluate evidence-based messaging about vaccinations that may be accepted by specific providers and their patients to appropriately dispel any misinformation. While the current results cannot make claims about the impact of information provision on exemption status, this is particularly important as pro-vaccine messaging may exacerbate vaccine hesitance and misperceptions among already hesitant parents in high-exemption regions [28].

As expected, the perceptions of responding exemptors about vaccination were more negative than those who reportedly did not exempt their children. Hesitation about vaccination safety and efficacy, as well as the trustworthiness of vaccination recommendations and vaccine-related information, were evident. These findings support previous literature indicating that the primary reasons for exemption are skepticism regarding the value of recommended vaccines [29–31], as well as a general distrust of vaccine information provided by sources such as the government, pharmaceutical industry, and certain providers in the medical community [31–34].

Risk perception about VPD also may influence an individual’s decision to exempt their children from vaccination. Responding exemptors more commonly reported knowing someone who had suffered from a VPD than non-exemptors. However, they also were less likely to report that the impacted individuals required any medical care or suffered severe manifestations of the disease. These results indicate that exemptors’ personal experience with VPD may support their belief that VPD are common, not serious, and that treatment is not necessary. The association between a belief that VPD are not severe and greater exemption rates in schools has been reported elsewhere among school personnel [25]. This is coupled with exemptors’ belief that immunity generated by infection is more robust than immunity generated from a vaccine. In addition to the findings regarding attitudes toward VPD, the safety and number of vaccines being administered overall, as well as the number administered in one clinic visit, were of particular concern to exemptors. These beliefs have been reported previously among school personnel at schools with high exemption rates [25]. Training school personnel about VPD severity and the importance of updating vaccination records may help to reduce the number of PBE forms offered to parents without their requesting them.

Systematic reviews of interventions aimed at clarifying misperceptions and/or decreasing parental vaccine hesitancy and refusal have yet to identify successful approaches for recommendation and/or to effectively evaluate their overall impact on hesitancy and vaccine uptake [35–37]. As mentioned, reasons for vaccine hesitancy are complex, vary widely, change over time and depend on a number of factors. Furthermore, interventions aimed at reducing misperceptions about vaccines and disease may be counter-productive and decrease intent to vaccinate [28]. Considering the lack of successful interventions and the context of our findings here, the great need for credibly-perceived sources and careful evaluation of messaging impact prior to intervention is again emphasized—including those delivered by providers [26,28,35]. For example, peer-peer education programs may be a target for intervention in high-exemption schools. However, while targeted and timely efforts communicating appropriate, evidence-based information are needed, much has been tried and the lack of effective interventions among vaccine-hesitant parents is of great concern [28,36,37].

Based on the results presented herein, in conjunction with other findings, changes were made by ADHS in July 2013 to the previous versions of all exemption forms requiring acknowledgment of the risks of exempting from each vaccination and as of May 2015, PBEs had declined slightly in Arizona [12]. Further work is needed to determine the long-term impact of the revised form. Since the revised PBE form was implemented, Arizona had measles cases associated with the highly publicized outbreak originating from California in December 2014. Seven of the 117 confirmed measles cases in 20 states and Washington, D.C were detected in Arizona [1]. The majority who contracted measles were unvaccinated [1]. As a result, in June, 2015, California joined Mississippi and West Virginia as the third state to adopt a medical-exemption-only regulation for school attendance; the California law took effect in July 2016 [38]. Given the lack of successful educational interventions to address vaccine hesitancy and vaccine uptake to-date it is possible that, in order to address geographic clusters of PBEs, Arizona may need to consider further changes to the current PBE process [39]. If PBE rate declines continue to be negligible and VPD rates continue to increase in Arizona, especially in high-exemption areas, legislative action to impose exemption-related processing fees or to adopt a medical-exemption-only regulation in Arizona may warrant consideration.

This study has limitations including the low response rate, lack of participation of all eligible schools, and the potential for bias in the participants who chose to respond. Thus, while our results share many similarities with previous reports on this topic, they are not generalizable to the overall population since we specifically targeted high-exemption schools in order to enrich the number of respondents. Even so, while the overall calculated response rates were low, the internal comparisons between responding exemptors (35%) and non-exemptors (65%) in these high-exemption schools should remain valid for informing future intervention strategies in these high PBE regions. While respondents may have been those with the strongest viewpoints on vaccination, we believe this reflects the actual population of Arizona, as those who participated in the survey were all among clusters of schools with higher than expected rates of PBEs, and all fit the demographic profiles of students attending high PBE schools (attended by predominantly white, higher-income students) [15]. Based on the results presented herein, willingness to participate in the survey was a factor which influenced our ability to compare differences in attitudes and perceptions among a greater number of exemptors and non-exemptors residing in high PBE clusters. Overall unwillingness to participate by non-respondents may have been influenced in part to the distrust of government and medical professionals cited by responding parents in these geographic regions. In addition, it is a conservative estimate of the response rate of individuals actually aware of the study. There may have been failed email addresses, incorrect filtering of the survey as spam, and other factors that led to lower awareness of the study. This study had limited funding and staff. The lack of available staff to track survey delivery to all parents and track participation, and the busy schedules of school administrators, were barriers for survey completion. As school administrators who are often highly burdened preferred to distribute the surveys, in the future, a study member or parent volunteer could distribute these instead to help keep track of which families indeed received and submitted the survey. Sending surveys and letters home with students to ensure delivery of the survey and increase study awareness could potentially increase willingness to participate among non-respondents and improve the response rate. Lastly, issues of record keeping may reflect vaccination coverage rates that conflict with reported PBE surveillance data in high-exemption schools [40].

## Conclusion

Based on the results reported herein, compared to non-exempting parent respondents, exemptors in Arizona high-exemption schools were more likely to report perceptions that VPDs are not severe and believe it is better for their child to develop immunity through illness rather than vaccination. Exempting parents were also less likely to trust physicians and information about vaccines, and were more likely to obtain their health care from a naturopath. Exempting respondents did not appear to have exempted their child from vaccination out of convenience but instead due to true personal beliefs. Within the limitation of a low response rate to the survey in this preliminary study, some general recommendations include the following for high-exemption schools in Arizona. First, there is a great need for development and provision of tailored educational materials and efforts that not only cite sources which the target audience trusts, but are also tested for impact prior to implementation. However, given the lack of successful interventions to-date, messaging to reduce PBE rates in high-exemption schools may be ineffective or counter-productive. If PBE and VPD rates continue to increase in high-exemption areas, resources to develop effective messaging for various populations and delivery methods may be unavailable. As states with easier nonmedical vaccine exemption policies are more likely to experience a VPD outbreak compared to those with more difficult policies [6–9], Arizona may need to consider other alternatives, such as more stringent requirements for PBEs and/or imposing processing fees to discourage high rates of PBEs. Considerable efforts remain to address the issue of PBEs in a comprehensive manner as the reasons for exemption appear to be multi-faceted. Further research and collaborative efforts among stakeholders are needed to address the issues outlined here.

## Supporting information

S1 Data Parent survey raw data. (XLSX)

S1 File Parent survey (English and Spanish versions). (PDF)

## Abbreviations

ADHS: Arizona Department of Health Services
PBE: Personal belief exemption
US: United States
VPD: Vaccine-Preventable Disease
