# Supplementary material for: Parental attitudes and perceptions associated with childhood vaccine exemptions in high-exemption schools
Source: PLoS One. 2018 Jun 14;13(6):e0198655. doi: 10.1371/journal.pone.0198655 (PMC6002085; doi:10.1371/journal.pone.0198655)
Supplement: S1 File — (PDF) [file pone.0198655.s002.pdf]

S1 File. Parent Survey (English and Spanish versions).

1. What language would you prefer to use for this survey?

- ◯ English
- ◯ Spanish

Dear parent or guardian,

Thank you for agreeing to complete this survey about childhood vaccinations. Please review the following letter for more information and/or if you have any questions. Your responses will help us to understand some of the concerns parents have regarding vaccinating their children.

At the completion of the survey, please follow the link to a page where you will provide your information. This information is not linked to your responses and is only collected to provide you with a small GIFT to thank you for your participation.

We value your opinion and THANK YOU for your time.

2. Have you ever delayed having your child get a shot for reasons other than illness or allergy?

- ◯ Yes
- ◯ No
- ◯ Don't Know

3. Have you ever decided not to have your child get a shot for reasons other than illness or allergy?

- ◯ Yes
- ◯ No
- ◯ Don't Know

4. If you had another infant today, would you want him/her to get all the recommended shots?

- ◯ Yes
- ◯ No
- ◯ Don't Know

5. On a scale from 0-10, how sure are you that following the recommended shot schedule is a good idea for your child?

0 - Not at all sure	1	2	3	4	5	6	7	8	9	10 - Completely sure
◯	◯	◯	◯	◯	◯	◯	◯	◯	◯	◯

6. On a scale of 1-5, where 1 is ‘strongly disagree’ and 5 is ‘strongly agree’, how much do you agree with the following statements? Check one per question.

	1 - Strongly disagree	2 - Disagree	3 - Not sure	4 - Agree	5 - Strongly agree
Children get more shots than are good for them.	○	○	○	○	○
I believe that many of the illnesses shots prevent are severe.	○	○	○	○	○
It is better for my child to develop immunity by getting sick than to get a shot.	○	○	○	○	○
It is better for children to get fewer shots at the same time.	○	○	○	○	○
I trust the information I receive about shots.	○	○	○	○	○
I am able to openly discuss my concerns about shots with my child’s doctor.	○	○	○	○	○

7. On a scale of 1-5, where 1 is not at all concerned and 5 is extremely concerned, rate your concern about the following. Check one per question.

	1 - Not at all concerned	2 - Not concerned	3 - Not sure	4 - Somewhat concerned	5 - Extremely concerned
How concerned are you that your child might have a serious side effect from a shot?	○	○	○	○	○
How concerned are you that any one of the childhood shots might not be safe?	○	○	○	○	○
How concerned are you that a shot might not prevent the disease?	○	○	○	○	○

8. Overall, how hesitant about childhood shots would you consider yourself to be?

Not at all hesitant	Not too hesitant	Not sure	Somewhat hesitant	Very hesitant
○	○	○	○	○

9. All things considered, how much do you trust your child’s doctor? (choose one or choose no doctor, if no doctor)

0 - Do not trust at all	1	2	3	4	5	6	7	8	9	10 - Completely trust	No doctor
○	○	○	○	○	○	○	○	○	○	○	○

10. What type of doctor do you visit?

- ○ M.D.
- ○ D.O.
- ○ Naturopath
- ○ I do not have a doctor

11. Have you ever known anyone who had a severe reaction to a shot? By severe we mean a reaction that made them get medical help.

- ○ Yes
- ○ No

12. Have you ever known anyone who got a disease that they could have gotten a shot for?

- ○ Yes, they were hospitalized
- ○ Yes, they needed a doctor
- ○ Yes, they didn't need a doctor
- ○ No

13. Have you ever taken a non-medical exemption for any or all of your children’s shots?

- ○ Yes
- ○ No

14. If you have taken a non-medical exemption for your children’s shots, what was your primary reason?

- ○ I lost my child’s shot records
- ○ I did not have time to go to the doctor to get updated shots
- ○ I do not believe my child should get shots

15. If you have taken a non-medical exemption for your children’s shots, how did you obtain it?

- ◯ I researched and downloaded a form from the Internet
- ◯ I requested the paperwork from the school office
- ◯ The school office offered the form without me asking
- ◯ I saw the forms sitting in the office

Questions about you and your family

16. What is your gender?

- ◯ Male
- ◯ Female

17. What is your age?

18. What is your race/ethnicity (check all that apply)

- ☐ White
- ☐ Black/African American
- ☐ Asian/Pacific-islander
- ☐ American Indian/Alaskan Native
- ☐ Hispanic or Latino
- ☐ Other (please specify)

19. What is your marital status?

- ◯ Married
- ◯ Never married
- ◯ Not married, but living with partner
- ◯ Divorced or separated
- ◯ Widowed

20. What is your total annual household income range?

- Less than $35,000
- $35,000-49,999
- $50,000-74,999
- $75,000-99,999
- $100,000 or greater

21. Are you employed?

- Yes
- No

22. If you have a spouse or partner living with you, are they employed?

- Yes
- No
- Not living with spouse or partner

23. What is the highest level of education you have completed?

- Less than 9th grade
- 9th to 12th grade
- High school graduate or GED
- Some college, but no degree
- Associate's degree
- Bachelor's degree
- Graduate or professional degree

24. What is the highest level of education for your spouse/partner living with you?

- ◯ No spouse/partner living with me
- ◯ Less than 9th grade
- ◯ 9th to 12th grade
- ◯ High school graduate or GED
- ◯ Some college, but no degree
- ◯ Associate's degree
- ◯ Bachelor's degree
- ◯ Graduate or professional degree

25. Do you have medical insurance that covers your child's shots?

- ◯ Yes, public insurance
- ◯ Yes, private insurance
- ◯ No

26. How many children live with you more than half the time?

[ ]

27. Do you have any comments about shots that are needed for your child to go to school?

[ ]

Estimado padre o tutor,

Gracias por aceptar a completar esta encuesta sobre las vacunas infantiles. Por favor revise la siguiente carta para obtener más información y / o si tiene alguna pregunta. Sus respuestas nos ayudarán a entender algunas de las preocupaciones de los padres con respecto a la vacunación de sus hijos.

Al final de la encuesta, por favor siga el enlace a una página donde se colecionará su información. Esta información no está vinculada a sus respuestas y sólo se colecte para ofrecerle un pequeño REGALO de agradecimiento por su participación.

Valoramos su opinión y GRACIAS por tu tiempo.

28. ¿Alguna vez ha retrasado que su hijo sea vacunado por razones que no sean por alguna enfermedad o alergia?

- Sí
- No
- No se

29. ¿Alguna vez ha decidido no dejar a que su hijo recibe una vacuna por razones que no sean por alguna enfermedad o alergia?

- Sí
- No
- No se

30. Si tuviera otro bebé hoy, ¿usted quisiera que él/ella obtuviera todas las vacunas recomendadas?

- Sí
- No
- No se

31. En una escala de 0-10, ¿qué tan seguro está de que seguir el programa de inyección recomendada es una buena idea para su hijo?

0 - Totalmente no seguro/a	1	2	3	4	5	6	7	8	9	10 - Completamente seguro/a
○	○	○	○	○	○	○	○	○	○	○

32. En una escala de 1-5, donde 1 es, "totalmente en desacuerdo" y 5 es, "muy de acuerdo", ¿que tanto estás de acuerdo con las siguientes afirmaciones?

	Totalmente en desacuerdo	No de acuerdo	No estoy segura/o	De acuerdo	Muy de acuerdo
Los niños reciben más vacunas de las cuales son buenas para ellos.	○	○	○	○	○
Creo que muchas de las enfermedades previnidas por las vacunas son graves.	○	○	○	○	○
Es major para mi hijo desarrollar inmunidad por enfermarse que por recibir una vacuna.	○	○	○	○	○
Es mejor que los niños reciben menos vacunas al mismo tiempo.	○	○	○	○	○
Confío en la información que recibo sobre las vacunas.	○	○	○	○	○
Soy capaz de hablar abiertamente de mis preocupaciones acerca de las vacunas con el médico de mi hijo.	○	○	○	○	○

33. En una escala de 1-5, donde 1 es, "no preocupada/o en absoluto" y 5 es, "extremadamente preocupada/o", marquee su preocupación por lo siguiente. Marque una por pregunta.

	No preocupada/o en absoluto	No me preocupo demasiado	No estoy segura/o	Mas o menos me preocupa	Extremadamente preocupada/o
¿Qué tan preocupada/o está de que su hijo pueda tener un grave efecto secundario por resulta de una vacuna?	○	○	○	○	○
¿Qué tan preocupada/o está de que cualquiera de las vacunas infantiles no podría ser seguro?	○	○	○	○	○
¿Qué tan preocupada/o está de que una vacuna no puede prevenir la enfermedad?	○	○	○	○	○

34. En general, ¿que tan vacilante sobre las vacunas infantiles te considerarías?

No vacilante en absoluto	No tan vacilante	No segura/o	Algo vacilante	Muy vacilante
○	○	○	○	○

35. A final de cuentas, ¿que tanto confía usted en el médico de su hijo? (marque uno o revise ningún médico, si no tiene médico)

0 - No confío en absoluto	1	2	3	4	5	6	7	8	9	10 - Completamente confío	Ningún médico
○	○	○	○	○	○	○	○	○	○	○	○

36. ¿Qué tipo de médico visita usted?

- ○ D.M.
- ○ D.O.
- ○ Naturópata
- ○ Ningún médico

37. ¿Alguna vez has conocido a alguien que ha tenido una reacción grave a resulto de una vacuna? Por grave se entiende que fue una reacción que les hizo buscar ayuda médica.

- ○ Sí
- ○ No

38. ¿Alguna vez has conocido a alguien que tuvo una enfermedad la cual podrían haber recibido una vacuna anteriormente?

- ○ Sí, ellos fueron hospitalizados
- ○ Sí, necesitaban un medico
- ○ Sí, no necesitaron un medico
- ○ No

39. ¿Alguna vez ha tomado una exención no médica para todas o algunas de las vacunas de sus hijos?

- ○ Sí
- ○ No

40. Si usted ha tomado una exención no médica para las vacunas de sus hijos, ¿cuál fue la razón principal?

- ◯ Perdí los registros de vacunación de mi hijo
- ◯ No tuve tiempo para ir al médico para recibir vacunas actualizadas
- ◯ Yo no creo que mi hijo debe vacunarse

41. Si usted ha tomado una exención no médica para las vacunas de sus hijos, ¿cómo la obtuvio?

- ◯ Lo investige y baje un formulario a través del Internet
- ◯ Yo pedí el papeleo de la oficina de la escuela
- ◯ La oficina de la escuela ofreció el formulario sin que lo solicitara
- ◯ Vi las formas accesibles en la oficina de la escuela

42. ¿Cuál es su sexo?

- ◯ Masculino
- ◯ Femenino

43. ¿Cuál es su edad?

[ ]

44. ¿Cuál es su raza/etnicidad? (marque todas que apliquen)

- [ ] Blanco
- [ ] Negro/Afro Americano
- [ ] Asiático/Islas del Pacífico
- [ ] Indio Americano/Nativo de Alaska
- [ ] Hispana/o u Latina/o
- [ ] Otro (por favor, especifique)

[ ]

45. Estado civil

- ◯ Casada/o
- ◯ Jamas casada/o
- ◯ No casada/o pero vivo con mi pareja
- ◯ Divorciada/o u separada/o
- ◯ Viuda/o

46. Ingreso familiar anual

- ◯ Menos de $35,000
- ◯ $35,000-49,999
- ◯ $50,000-74,999
- ◯ $75,000-99,999
- ◯ $100,000 o más

47. ¿Está usted trabajando?

- ◯ Sí
- ◯ No

48. Si usted tiene un cónyuge o pareja que vive con usted, ¿están trabajando?

- ◯ Sí
- ◯ No
- ◯ No cónyuge o pareja que vive con usted

49. ¿Cuál es su nivel educativo más alto que se haya completado?

- ◯ Menos de 9º grado
- ◯ 9º al 12º grado
- ◯ Graduada/o de preparatoria / GED
- ◯ Algunos estudios universitarios, pero sin título
- ◯ Diploma de asociado/titulo a nivel medio
- ◯ Diploma de Bachiller/tecnico
- ◯ Licenciatura o profesional

50. ¿Cuál es el nivel más alto de educación de su cónyuge / pareja que vive con usted?

- ◯ No tengo cónyuge / pareja viviendo conmigo
- ◯ Menos de 9º grado
- ◯ 9º al 12º grado
- ◯ Graduada/o de preparatoria / GED
- ◯ Algunos estudios universitarios, pero sin título
- ◯ Diploma de asociado/titulo a nivel medio
- ◯ Diploma de Bachiller/tecnico
- ◯ Licenciatura o profesional

51. ¿Tiene seguro médico que cubra las vacunas de su hijo?

- ◯ Sí, seguro público
- ◯ Sí, seguro privado
- ◯ No

52. ¿Cuántos niños viven con usted más de la mitad del tiempo?

53. ¿Tiene algún comentario sobre las vacunas que se requieren para que su hijo vaya a la escuela?

Esto completa la encuesta. Muchas gracias por su participación. Para mostrar nuestro agradecimiento, por favor siga el enlace a continuación para poder enviarle un pequeño regalo de agradecimiento. Le recordamos que su identidad se mantendrá completamente separada y no conectariamos esta información en ninguna manera con las respuestas que ha proporcionado en la encuesta.

Gracias.

Haga clic aquí o copie **https://www.surveymonkey.com/s/datos** a tu navegador.

This completes the survey. Thank you very much for your participation. To show our appreciation, please follow the link below so we may send you a small gift of appreciation. Please note that your identity will be kept completely separate and we will not connect this information in any way to the answers provided in the survey.
Thank you.
Click here or copy **https://www.surveymonkey.com/s/rewardinfo to your browser.**
